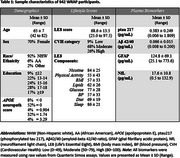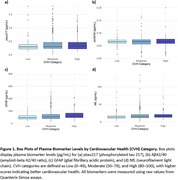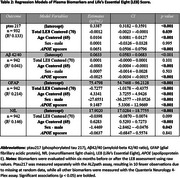# Life's Essential Eight and Dementia‐Related Plasma Biomarkers: An Exploratory Analysis

**DOI:** 10.1002/alz70860_107538

**Published:** 2025-12-23

**Authors:** Diandra N. Denier‐Fields, Maisha Islam, Nathaniel A. Chin, Ramiro Eduardo Rea Reyes, Rachael E. Wilson, Erin M. Jonaitis, Rebecca E. Langhough, Sterling C Johnson, Corinne D. Engelman

**Affiliations:** ^1^ University of Wisconsin‐Madison, Madison, WI, USA

## Abstract

**Background:**

The American Heart Association (AHA) Life's Essential Eight (LE8) measures eight lifestyle factors contributing to cardiovascular health, including nicotine exposure, physical activity, diet, body mass index (BMI), blood lipids, blood sugar, blood pressure, and sleep. LE8 provides a granular scoring system (0–100 points per factor, averaged for a total score), reflecting incremental changes and medication use. Poor cardiovascular health has been linked to cognitive decline and dementia, and blood‐based biomarkers are increasingly used to assess dementia risk. Plasma biomarkers such as phosphorylated tau 217 (ptau217) and amyloid‐beta 42/40 (Aβ 42/40) are associated with Alzheimer's disease (AD), while glial fibrillary acidic protein (GFAP) and neurofilament light chain (NfL) indicate non‐specific neuroinflammation and neurodegeneration, respectively. This study examined LE8's associations in late life with concurrent AD‐related plasma biomarkers.

**Method:**

LE8 scores were calculated for 942 participants in the Wisconsin Registry for Alzheimer's Prevention (WRAP) cohort who were free of dementia at baseline. Scores were centered at 70, which closely approximates the observed mean of 68.6 and matched to plasma biomarkers measured within 6 months before or after assessment. General linear models evaluated the relationships between LE8 scores and biomarkers, adjusting for age at LE8, sex, and apolipoprotein E (*APOE*).

**Result:**

Sample characteristics are summarized in Table 1. LE8 scores ranged from 25 to 97.5, with higher scores indicating better cardiovascular health. Figure 1 illustrates biomarker distributions across cardiovascular health categories. Regression models (Table 2) showed that LE8 scores above 70 were significantly associated with lower concentrations of ptau217 (β = ‐0.0012, *p* = 0.039) and GFAP ( β = ‐0.7277, *p* = <0.001) per point increase, suggesting a potential role of lifestyle in modulating AD‐related plasma biomarkers. No associations were observed with Aβ42/40 or NfL.

**Conclusion:**

These findings suggest that better cardiovascular health, as measured by LE8, may influence circulating levels of certain AD‐related biomarkers. Further investigation is needed to assess whether these associations persist over time and extend to cognitive decline and structural brain changes associated with dementia pathology. Future analyses will incorporate longitudinal data to explore these relationships, providing critical insights into the role of lifestyle in dementia prevention and progression.